# The Activity of FDA-Approved Prodrug Isavuconazonium Sulfate and Its Major Metabolite Isavuconazole Against *Naegleria fowleri*

**DOI:** 10.3390/pharmaceutics18010103

**Published:** 2026-01-12

**Authors:** Hayley Fong, Anjan Debnath

**Affiliations:** Center for Discovery and Innovation in Parasitic Diseases, Skaggs School of Pharmacy and Pharmaceutical Sciences, University of California San Diego, 9255 Pharmacy Lane, MC 0657, La Jolla, CA 92093-0657, USA

**Keywords:** *Naegleria*, primary amoebic meningoencephalitis, parasite, free-living amoeba, CY51 inhibitors, isavuconazole, isavuconazonium, synergy

## Abstract

**Objectives**: Free-living amoeba *Naegleria fowleri* causes primary amoebic meningoencephalitis (PAM). While infection is rare, PAM’s fatality rate exceeds 97%. The recommended treatment includes combination therapy, which does not result in uniform survival. Thus, there is a critical unmet need for finding better therapy for PAM. Drug repurposing can expedite the discovery of effective treatment for PAM. Isavuconazonium is approved for the treatment of fungal infections. Given that isavuconazole is the major metabolite of isavuconazonium and isavuconazole penetrates into the brain with high efficiency, our objective was to determine the activity of both isavuconazonium and isavuconazole on *N. fowleri* trophozoites. **Methods**: To test the effect of both compounds, we determined their dose–responses against *N. fowleri* and two mammalian cells. To establish how fast the prodrug and the metabolite kill the trophozoites, we measured potency at different time points. Finally, we investigated the effect of combining isavuconazonium or isavuconazole with amphotericin B on both *N. fowleri* and mammalian cells. **Results**: Both isavuconazonium and the metabolite isavuconazole were active against multiple strains, with clinically relevant isavuconazole exhibiting potency ranging between 0.1 and 0.6 µM. They were less toxic on mammalian cells. Isavuconazonium and isavuconazole required 24 h to achieve nanomolar potency. Combination with amphotericin B was synergistic without eliciting toxicity on mammalian cells. **Conclusions**: Our findings, together with the use of intravenous and oral formulations of isavuconazonium to treat pediatric and adult patients, support further in vivo efficacy study of isavuconazonium for its potential use for the treatment of PAM.

## 1. Introduction

*Naegleria fowleri* is an opportunistic and pathogenic single-celled free-living amoeba. This organism exists in one of three interchangeable morphological life stages: cyst, trophozoite, and flagellated form [1]. Active and infectious trophozoites are found in warm natural freshwater sources (lakes, ponds, and rivers) or improperly treated water sources (splash pads and swimming pools) [2]. Infection of *N. fowleri* leads to primary amoebic meningoencephalitis (PAM). Between 1962 and 2023, there were 488 cases of PAM reported globally [3], of which 157 cases occurred in the United States [4]. While the occurrence in the overall population is rare, the mortality rate of PAM exceeds 97% [5].

Symptoms of PAM manifest between 1 and 12 days post-exposure (median of 5 days) and are often non-specific: fever, headache, nausea, and fatigue. The severity of the symptoms rapidly increases with mental status alterations and seizures, subsequently resulting in coma and death. Due to the rarity of PAM, many patients are misdiagnosed and treated for bacterial or viral meningitis, with the actual cause of death discovered post-mortem [6].

The treatment guidance set by the US Centers for Disease Control and Prevention (CDC) is based on survivor case reports or in vitro studies due to the high mortality and rarity of *N. fowleri* infection [7]. The current recommendation consists of conventional amphotericin B (intravenous), miltefosine (oral), fluconazole or another azole-containing compound (intravenous or oral), rifampin (intravenous or oral), azithromycin (intravenous or oral), and dexamethasone (intravenous) [8]. Amphotericin B is widely considered the drug of choice for treatment of PAM, but only 19% of PAM patients treated with amphotericin B survived [9]. Moreover, amphotericin B produces high nephrotoxicity due to severe vasoconstriction that decreases renal blood flow [10]. With these limitations, it is critical to find safer and more efficient drugs to treat this overwhelmingly fatal disease.

**Figure 1:**
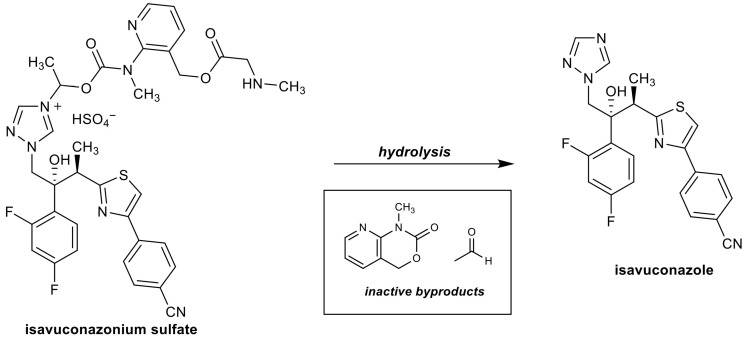
Esterase-mediated hydrolysis of the prodrug isavuconazonium to its active form, isavuconazole.

Earlier, we demonstrated anti-*N. fowleri* activity of isavuconazole on a reference strain [11], but no study was conducted to determine its activity on different strains of *N. fowleri*, nor was it known if isavuconazole could be combined with amphotericin B to enhance its amoebicidal activity. Moreover, isavuconazole is the active metabolite of the prodrug isavuconazonium sulfate (Cresemba^®^), which is clinically approved for both pediatric and adult patients in the treatment of fungal infections caused by invasive aspergillosis and invasive mucormycosis. Although plasma esterase enzymes, particularly butyrylcholinesterase, metabolize isavuconazonium into isavuconazole [12] (Figure 1), the activity of isavuconazonium sulfate, either alone or in combination with amphotericin B, was not determined. In this study, we explored the activity of both the prodrug isavuconazonium and the active metabolite isavuconazole against multiple genotypes of *N. fowleri* trophozoites. We also investigated the combined trophocidal activity of the prodrug and its active moiety with the recommended drug, amphotericin B, and evaluated the morphological effect of the synergistic activity of the drugs on the trophozoites.

## 2. Materials and Methods

### 2.1. N. fowleri Cell Culture

*N. fowleri* trophozoites of European KUL (ATCC 30808), US genotypes I and II (Davis and CAMP, respectively), and Australian CDC:V1005 were cultivated in an axenic condition in Nelson’s medium supplemented with 10% fetal bovine serum and maintained at 37 °C with 5% CO_2_ [13]. Strains from US and Australia were acquired from the CDC (Atlanta, GA, USA). Experiments were performed using *N. fowleri* trophozoites harvested in the logarithmic phase of growth (48 h). Counting of trophozoites was conducted using a hemocytometer.

### 2.2. Mammalian Epithelial Cell Culture

Human embryonic kidney cells (HEK293) and human colon cancer cells (HT-29) were cultured in Dulbecco’s Modified Eagle Medium supplemented with penicillin (100 U/mL), streptomycin (100 µg/mL), and 10% fetal bovine serum [14]. Experiments using human cells were performed during the logarithmic phase of growth. Cells were counted using a hemocytometer.

### 2.3. In Vitro Activity of Isavuconazonium Sulfate and Isavuconazole Against Different Genotypes of N. fowleri

Isavuconazonium sulfate is highly soluble in aqueous solutions with a solubility >1000 mg/mL [15]. Stock solutions of 10 mM isavuconazonium sulfate (MedChem Express, Monmouth Junction, NJ, USA) and amphotericin B (GoldBio, St Louis, MO, USA) in 100% DMSO were stored at −20 °C. Isavuconazole has a low aqueous solubility and its solubility in aqueous buffers ranges from 5.3 to 23 µg/mL [16]. A stock solution of 5 mM isavuconazole (Santa Cruz Biotechnology, Dallas, TX, USA) was prepared in aqueous 40% (*w*/*v*) sulfobutylether-β-cyclodextrin (SBE-β-CD) (MedChem Express) by sonication at room temperature at 40 Hz for up to 10 min, and pH was adjusted to 7 using dilute sodium hydroxide solution. A total of 16 concentrations of isavuconazonium sulfate, isavuconazole, and amphotericin B were tested against KUL, CAMP, Davis, and CDC:V1005 strains of *N. fowleri*. A 2-fold serial dilution was performed with each 5- or 10 mM stock concentration in a 96-well round-bottom dilution plate. The appropriate vehicle—100% DMSO or 40% (*w*/*v*) SBE-β-CD—was used to generate a concentration range of 5 or 10 mM to 0.0002 or 0.0003 mM, respectively. A total of 0.5 µL of each concentration was transferred in triplicate into a white, 96-well flat-bottom microplate (Greiner BioOne, Monroe, NC, USA) followed by addition of *N. fowleri* trophozoites at 10^5^/mL in a final volume of 100 µL/well. The final set of 16-point concentrations ranged from 25 to 0.0008 µM in 40% SBE-β-CD or 50 to 0.0008 or 0.0015 μM in 100% DMSO [11,13]. In addition, 0.5% (*v*/*v*) DMSO or 0.5% (*v*/*v*) 40% (*w*/*v*) SBE- β-CD was used as a negative or vehicle control and 50 µM amphotericin B was used as a positive or trophocidal control. Assay plates were incubated for 48 h at 37 °C.

The trophocidal effect of the compounds was measured using 25 µL of CellTiter-Glo Luminsecent Viability Assay reagent (Promega, Madison, WI, USA) dispensed into each well of the plate following a 10 min equilibration at room temperature. CellTiter-Glo reagent induced cell lysis when the plates were kept on an orbital shaker at 300 rpm for 10 min at room temperature. Oxidation of luciferin, prompted by the release of intracellular ATP, emits luminescence, which was allowed to stabilize for 10 min at room temperature. ATP bioluminescence of the trophozoites in each plate was then measured using an Envision 2104 Multilabel Reader (PerkinElmer, Waltham, MA, USA). Data from a minimum of three independent biological experiments were used to determine EC_50_ values on GraphPad Prism 10. Sigmoidal dose–response curves were generated and statistical parameters including 95% confidence interval and goodness of fit (R^2^) were determined.

Viability of *N. fowleri* KUL trophozoites incubated with different concentrations of one of the compounds was also determined by the trypan blue exclusion method [17]. A total of 5 × 10^4^ trophozoites were incubated in triplicate with different concentrations of isavuconazole ranging from 0.1 to 0.78 µM in a 24-well tissue culture plate in a total volume of 0.5 mL for 48 h at 37 °C. 40% SBE-β-CD-treated trophozoites were used as a control. Cells were counted by hemocytometer after 48 h following the addition of trypan blue. The percentage of viable trophozoites at different concentrations of isavuconazole was calculated and data from three independent biological experiments were used to plot the results on GraphPad Prism 10.

### 2.4. Evaluation of Mammalian Cytotoxicity of Isavuconazonium and Isavuconazole

Since the intestine is involved in the absorption and first-pass metabolism of drugs [18] and the kidney plays a role in the metabolism and elimination of drugs [19], we selected both human HT-29 intestinal and HEK293 kidney cells for cytotoxicity studies. Mammalian cytotoxicity assays were performed in white, 96-well screen plates (Greiner BioOne) by adding HEK293 or HT-29 at a concentration of 10^5^/mL with 0.5% (*v*/*v*) vehicle control or compound for a final volume of 100 µL/well. Compounds were tested in triplicate between 0.006 and 200 µM. Staurosporine (MedChem Express) at 100 µM was used as the positive cytotoxic control. Cell viability was measured after 72 h of incubation at 37 °C using the CellTiter-Glo Luminescent Cell Viability Assay [14]. Sigmoidal dose–response curves were generated and statistical parameters including 95% confidence interval and R^2^ were determined.

### 2.5. Effect of Isavuconazonium and Isavuconazole on Growth Inhibition at Different Time Points

The relative rate at which isavuconazonium sulfate and isavuconazole exhibit activity against *N. fowleri* was measured using the KUL strain at different time points. The KUL strain was incubated with isavuconazonium sulfate (from 0.39 to 50 µM) and isavuconazole (from 0.2 to 25 µM) for 10, 16, and 24 h in 96-well microplates. The growth inhibition percent and EC_50_ at these time points were determined by using the CellTiter-Glo Luminsecent Cell Viability Assay following the same protocol as described above [14]. Assays were performed in triplicate in three independent biological replicates.

### 2.6. Effect of Combination of Isavuconazonium or Isavuconazole and Amphotericin B on N. fowleri

To determine the combined effect of isavuconazonium sulfate and amphotericin B against *N. fowleri*, two-fold dilutions of isavuconazonium sulfate and amphotericin B were prepared from a stock of 1.25 mM amphotericin B and 0.625 mM isavuconazonium sulfate. A volume of 0.25 μL of each diluted drug was transferred into an opaque-bottom tissue culture 96-well plate (Greiner BioOne) in a matrix way. *N. fowleri* trophozoites (99.5 μL, 10^4^ amoebae) were added to yield a final volume of 100 μL per well, reaching different ratios (1:1, 1:2, 1:4, 1:8, 1:16, 2:1, 4:1, and 8:1) in the combination matrix formed by the mixtures of amphotericin B from 3.125 to 0.024 μM in rows and isavuconazonium sulfate from 1.56 to 0.012 μM in columns. Equal concentrations of amphotericin B and isavuconazonium sulfate were parallelly and independently evaluated for activity [20]. 0.5% (*v*/*v*) DMSO and 50 µM amphotericin B were used as negative and positive controls, respectively. All experiments were performed in triplicate. *N. fowleri* KUL trophozoites were incubated with the drug combinations for 48 h at 37 °C. Assay plates were equilibrated to room temperature for 10 min before quantifying the growth inhibition percentage using the CellTiter-Glo Luminescent Viability Assay.

An equivalent procedure was performed with isavuconazole in 40% SBE-β-CD, with the noted differences as follows: 1.25 mM isavuconazole stock solution was used in the matrix to combine amphotericin B with isavuconazole from 3.125 to 0.024 µM in both columns and rows. SBE-β-CD (0.5%) was used as a negative control.

The percentage of growth inhibition was calculated, and the effect of the drug combinations was determined by CompuSyn software (Version 1.0) following the Chou–Talalay method [21]. The software calculated the combination index (CI) values for additive (CI = 1), antagonistic (CI > 1), or synergistic (CI < 1) effect [21].

### 2.7. Microscopic Evaluation of Combining Isavuconazonium or Isavuconazole with Amphotericin B

The calculated synergistic effect of isavuconazonium sulfate or isavuconazole with amphotericin B was evaluated experimentally with one of the synergistic ratios in 96-well clear-bottom plates. Trophozoites (10^4^ *N. fowleri* per well) were incubated with 0.2 µM of isavuconazonium and 0.05 µM of amphotericin B (4:1), or 0.1 µM of isavuconazole and 0.05 µM of amphotericin B (2:1), both alone and in combination for 48 h. Trophozoites were treated with 0.5% DMSO or 0.5% SBE-β-CD as a negative control. All experiments were conducted in triplicate. Images of trophozoites were captured at 48 h using a Zeiss Axiovert 40 CFL phase contrast microscope (Carl Zeiss, White Plains, NY, USA).

### 2.8. Viability Scoring of Trophozoites Treated with a Combination of Isavuconazonium or Isavuconazole and Amphotericin B at a Synergistic Ratio

Viability of KUL trophozoites incubated with one of the synergistic ratios for a combination of isavuconazonium and amphotericin B (4:1) and isavuconazole and amphotericin B (2:1) was also determined by the trypan blue exclusion method. A total of 5 × 10^4^ trophozoites were incubated in triplicate with 0.2 µM of isavuconazonium, 0.05 µM amphotericin B, and a combination of 0.2 and 0.05 µM of isavuconazonium and amphotericin B, respectively, in a 24-well tissue culture plate for 48 h at 37 °C. Similarly, an experiment was performed in triplicate with 0.1 µM of isavuconazole, 0.05 µM amphotericin B, and a combination of 0.1 and 0.05 µM of isavuconazole and amphotericin B, respectively, for 48 h at 37 °C. 0.5% DMSO- or 0.5% SBE-β-CD-treated trophozoites were used as controls. Cells were counted by hemocytometer after 48 h following the addition of trypan blue. The percentage of viable trophozoites incubated with isavuconazonium or isavuconazole and amphotericin B alone and in combination with two compounds was calculated and data from three independent biological experiments were used to plot the results on GraphPad Prism 10.

### 2.9. Evaluation of Mammalian Cytotoxicity of Combination of Isavuconazonium or Isavuconazole with Amphotericin B

Mammalian cytotoxicity assays were performed in 96-well screen plates. A total of 0.5 μL of the compound was transferred into a well followed by the addition of HEK293 or HT-29 cells (10^4^ cells) in a total volume of 100 μL/well. This resulted in concentrations of 1.2 μM of isavuconazonium, 0.2 µM of isavuconazole, and 0.3, 0.1, and 0.05 µM of amphotericin B alone; a combination of 1.2 µM of isavuconazonium with 0.3 µM of amphotericin B (4:1); a combination of 0.2 µM of isavuconazole and 0.1 µM of amphotericin B (2:1); and a combination of 0.2 µM of isavuconazole with 0.05 µM of amphotericin B (4:1). A final vehicle concentration of 0.5% (*v*/*v*) was used as a negative control and 100 μM staurosporine served as a positive control. The assay was conducted in triplicate at 37 °C for 72 h. Subsequently, cell viability was measured using the CellTiter-Glo Luminescent Cell Viability Assay [14].

## 3. Results

### 3.1. In Vitro Activity of Isavuconazonium Sulfate and Isavuconazole Against Different Genotypes of N. fowleri and Two Mammalian Cells

Based on our previous demonstration [11] of anti-*N. fowleri* activity by various conazoles including isavuconazole, we determined the activity of the prodrug isavuconazonium sulfate against four strains of *N. fowleri*. The dose–response curves for both isavuconazonium and isavuconazole against different strains of *N. fowleri* provided R^2^ values between 0.9 and 1. While isavuconazonium was equipotent to amphotericin B with an EC_50_ of 0.1 µM on the European KUL strain, the EC_50_ values of isavuconazonium on US strains Davis and CAMP and the Australian strain CDC:V1005 were 1.9, 3.8, and 4.7 µM, respectively (Table 1). Isavuconazonium provided selectivity indices of about 150–400 when tested on two mammalian cell lines, HEK293 and HT-29, for 72 h (Table 2).

Since isavuconazole is the active moiety of the FDA-approved prodrug isavuconazonium sulfate that demonstrated nanomolar potency against the reference strain KUL, we determined the activity of isavuconazole against clinical strains including KUL, Davis, CAMP, and CDC:V1005. Although isavuconazole was dissolved in SBE-β-CD for better solubility and this solvent was used in the follow-up experiments, isavuconazole, when dissolved in DMSO, did not cause any difference in the EC_50_ value against the reference KUL strain (Figure S1). Isavuconazole retained nanomolar potency against all strains tested (Table 1) and provided selectivity indices of about 35–200 or 50–300 based on the strain of *N. fowleri* and the mammalian cell types examined (Table 2).

**Figure 2:**
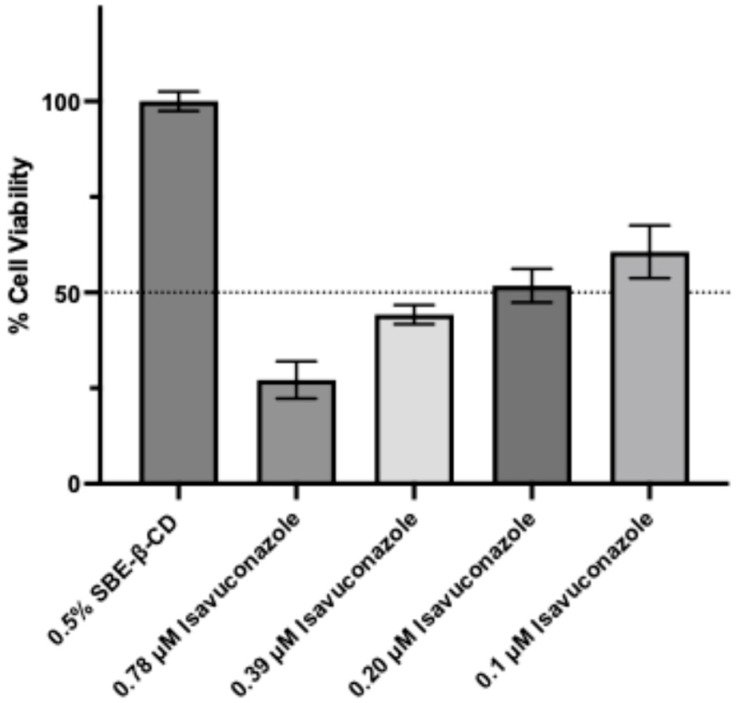
Viability of *N. fowleri* KUL trophozoites in the presence of isavuconazole, as determined by the trypan blue exclusion method. Trophozoites were treated with different concentrations of isavuconazole for 48 h and cell viability was determined by staining the cells with trypan blue and counting the trophozoites by hemocytometer. Values plotted are from the mean ± standard error of mean (SEM) of cells incubated with 0.5% SBE-β-CD control and isavuconazole. The dashed line represents 50% cell viability.

Trypan blue exclusion assay with a compound isavuconazole further confirmed dose-dependent cell viability of *N. fowleri* trophozoites. While a higher concentration (0.78 µM) of isavuconazole led to about 70% cell death, 52 ± 3.1 and 61 ± 4.9% cell viability was observed with 0.2 and 0.1 µM isavuconazole (Figure 2). Although trypan blue exclusion assay is less sensitive than the CellTiter-Glo Luminescent Viability Assay, the result is consistent with the EC_50_ value determined by the CellTiter-Glo Luminescent Viability Assay.

### 3.2. Effect of Isavuconazonium and Isavuconazole on Growth Inhibition at Different Time Points

**Figure 3:**
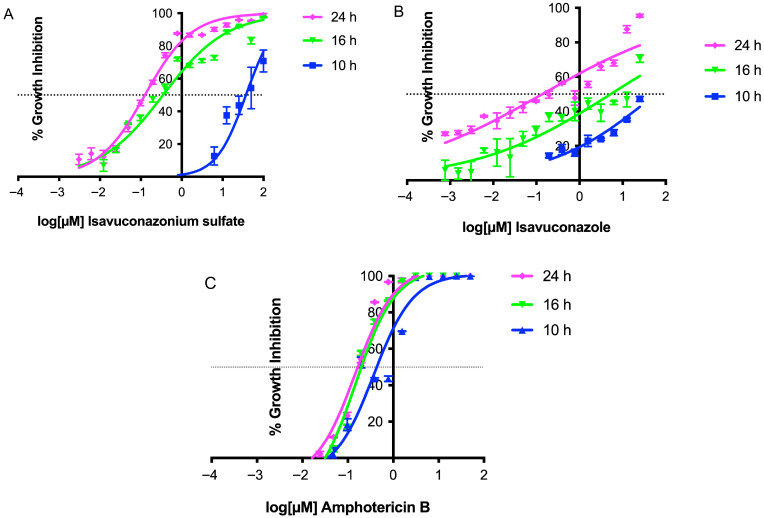
EC_50_ curves of isavuconazonium sulfate, isavuconazole, and amphotericin B at 10, 16, and 24 h against *N. fowleri*. KUL trophozoites were treated with different concentrations of compounds in triplicate and growth inhibition curves were generated from mean ± SEM of (**A**) isavuconazonium, (**B**) isavuconazole, and (**C**) amphotericin B. Dashed line represents 50% growth inhibition.

The effect of the trophocidal activity of isavuconazonium sulfate and isavuconazole against *N. fowleri* was determined by measuring the EC_50_ against the KUL strain at 10, 16, and 24 h of incubation with the compounds. The effect was compared to the dose–response (EC_50_) of amphotericin B at the same time points. The EC_50_ curves generated for the KUL strain at 10, 16, and 24 h demonstrated that both isavuconazonium (Figure 3A) and isavuconazole (Figure 3B) required 24 h to reach the EC_50_ value of 0.1 µM. Growth inhibition of trophozoites by amphotericin B required 16 h to reach its EC_50_ of 0.2 µM (Figure 3C).

### 3.3. Effect of Combination of Isavuconazonium or Isavuconazole and Amphotericin B on N. fowleri

Since all treatment protocols for PAM that resulted in the survival of patients contained amphotericin B [23], we investigated the effect of combining isavuconazonium and its major metabolite isavuconazole with amphotericin B. We measured the growth inhibitory effects of isavuconazonium–amphotericin B and isavuconazole–amphotericin B pairings at fixed concentration ratios. We used CompuSyn software [21] to assess the dose–effect relationships between the two drugs and the software generated isobolograms to calculate CI and dose reduction indices. A 97% growth inhibition with about a 7- to 20-fold dose reduction for isavuconazonium and 3- to 4-fold dose reduction for amphotericin B was achieved at 2:1 (CI = 0.4), 4:1 (CI = 0.4), and 8:1 (CI = 0.4) ratios of isavuconazonium and amphotericin B (Table 3 and Figure S2).

Like isavuconazonium, a 97% growth inhibition of trophozoites with about a 200- to 400-fold dose reduction for isavuconazole and about a 5- to 10-fold dose reduction for amphotericin B was achieved at 2:1 (CI = 0.3), 4:1 (CI = 0.2), and 8:1 (CI = 0.2) ratios of isavuconazole and amphotericin B (Table 4 and Figure S3).

### 3.4. Microscopic Evaluation of Combining Isavuconazonium or Isavuconazole with Amphotericin B

**Figure 4:**
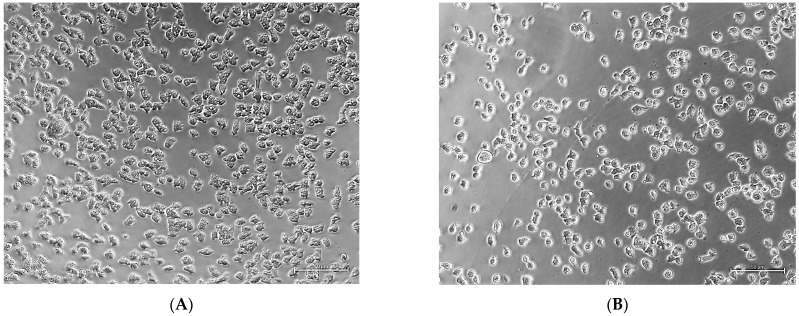

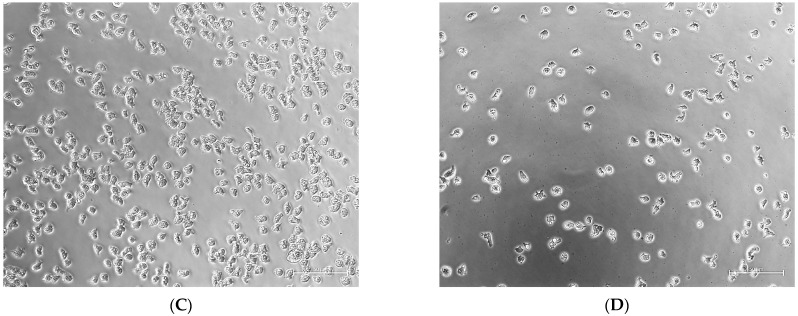
The synergistic effect of isavuconazonium sulfate and amphotericin B. The images in a phase contrast microscope show KUL trophozoites incubated for 48 h with (**A**) 0.5% DMSO, (**B**) 0.2 µM isavuconazonium, (**C**) 0.05 µM amphotericin B, and (**D**) a combination of 0.2 μM of isavuconazonium and 0.05 µM of amphotericin B. The cells treated with a combination of drugs are much smaller in size and could not grow, whereas DMSO-treated cells are amoeboid-shaped with visible cytoplasm. The scale bar in the bottom right of each picture represents 50 µm.

To confirm the synergistic effect calculated by CompuSyn, we performed experiments for microscopic evaluation of trophozoites when combined with one of the synergistic ratios as determined by CompuSyn. Our phenotypic experiments with a combination of 0.2 µM isavuconazonium and 0.05 µM amphotericin B (4:1) confirmed the enhanced activity of the partnering of the two drugs compared to the activity of the drugs when used alone. While the combined effect of the two drugs led to smaller-sized rounded trophozoites (Figure 4D), the cells treated with DMSO (Figure 4A) or one compound alone were amoeboid-shaped with visible cytoplasm (Figure 4B,C).

**Figure 5:**
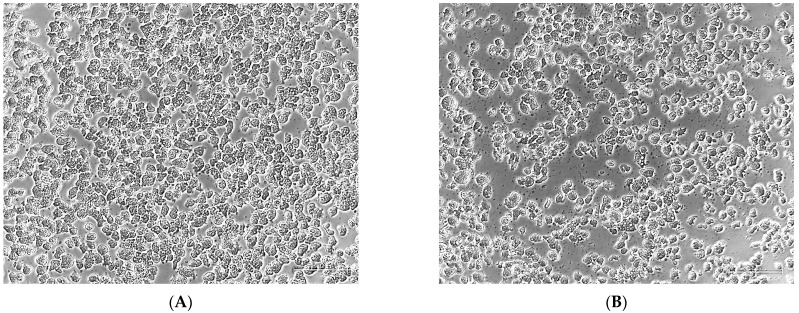

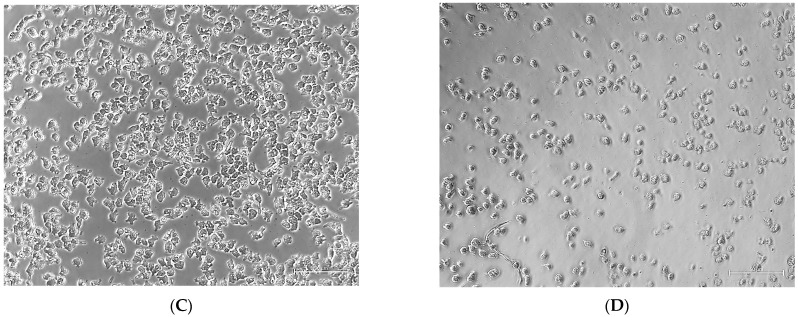
The synergistic effect of isavuconazole and amphotericin B. The images in a phase contrast microscope show KUL trophozoites treated for 48 h with (**A**) 0.5% SBE-β-CD, (**B**) 0.1 µM isavuconazole, (**C**) 0.05 µM amphotericin B, and (**D**) a combination of 0.1 μM of isavuconazole and 0.05 µM of amphotericin B. The cells treated with a combination of compounds are much smaller in size and could not grow, whereas SBE-β-CD-treated cells are amoeboid-shaped with visible cytoplasm. The scale bar in the bottom right of each picture represents 50 µm.

A similar effect was also observed when trophozoites were incubated with a combination of amphotericin B and isavuconazole, the major metabolite of isavuconazonium. The effect of a combination of 0.1 µM of isavuconazole and 0.05 µM of amphotericin B (2:1) (Figure 5D) was more pronounced on the morphology of trophozoites than the effect of SBE-β-CD (Figure 5A) or each of the compounds alone (Figure 5B,C).

### 3.5. Viability Scoring of Trophozoites Treated with a Combination of Isavuconazonium or Isavuconazole and Amphotericin B at a Synergistic Ratio

**Figure 6:**
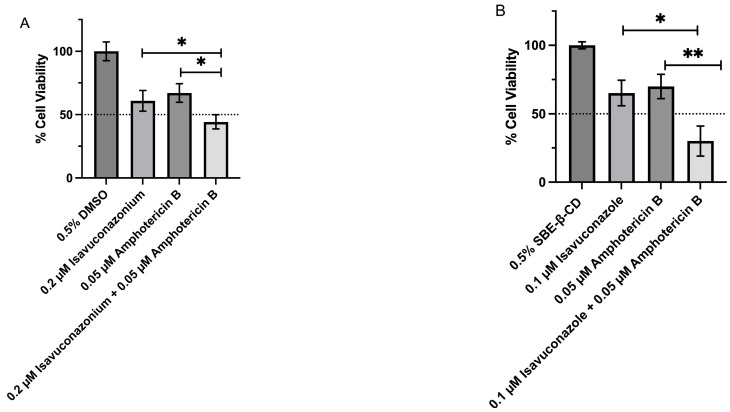
Viability of *N. fowleri* KUL trophozoites in the presence of a combination of isavuconazonium or isavuconazole and amphotericin B, as determined by the trypan blue exclusion method. Trophozoites were treated with (**A**) 0.2 µM isavuconazonium alone, 0.05 µM amphotericin B alone, a combination of 0.2 µM of isavuconazonium and 0.05 µM of amphotericin B, and 0.5% DMSO vehicle control, and (**B**) 0.1 µM isavuconazole alone, 0.05 µM amphotericin B alone, a combination of 0.1 µM of isavuconazole and 0.05 µM of amphotericin B, and 0.5% SBE-β-CD vehicle control for 48 h, and cell viability was determined by staining the cells with trypan blue and counting the trophozoites by hemocytometer. The values plotted are from the mean ± SEM of viable cells and the dashed line represents 50% cell viability. * *p* < 0.05, ** *p* < 0.01 by Student’s *t*-test compared to a single-drug-treated *N. fowleri*.

Following microscopic confirmation of the synergistic effect of a combination of isavuconazonium and amphotericin B at 4:1, and isavuconazole and amphotericin B at 2:1, we further validated the synergistic activity of these two compounds by trypan blue exclusion assay for quantitative assessment of the viability of trophozoites. While a single compound concentration of 0.2 µM of isavuconazonium and 0.05 µM of amphotericin B resulted in 60.9 ± 4.7 and 67 ± 4.2% cell viability, respectively, a combination of these two compounds at the same concentration elicited a significant (*p* < 0.05) reduction in the viability of trophozoites (44.3 ± 3.3%) compared to trophozoites treated with one drug alone (Figure 6A). Similarly, while treatment with a single drug of 0.1 µM of isavuconazole or 0.05 µM of amphotericin B exhibited cell viability of 65.2 ± 5.4 and 69.9 ± 5%, respectively, a significant (*p* < 0.05) reduction in cell viability (30.1 ± 6.4%) was observed when both drugs were combined at the same concentration (Figure 6B).

### 3.6. Evaluation of Mammalian Cytotoxicity of Combination of Isavuconazonium or Isavuconazole with Amphotericin B

Since the combination of isavuconazonium and amphotericin B at a ratio of 4:1 provided synergistic activity against *N. fowleri* trophozoites, we tested the activity of these two drugs at a ratio of 4:1 on two mammalian cells, HEK293 kidney cells and HT-29 intestinal cells. A combination of 1.2 µM isavuconazonium and 0.3 µM amphotericin B caused only about 6 ± 1.2% growth inhibition of HEK293 and about 14.3 ± 5.6% growth inhibition of HT-29 cells. The percentage of growth inhibition on these two cells was not significant (*p* > 0.05) compared to 0.5% DMSO-treated control cells.

Similarly, we selected a synergistic combination of isavuconazole and amphotericin B at 2:1 to conduct a cytotoxicity experiment on HEK293 and HT-29 cells. A combination of 0.2 µM isavuconazole and 0.1 µM amphotericin B (2:1) caused no growth inhibition of HEK293 and 2.8 ± 0.8% growth inhibition of HT-29 cells. This ratio of isavuconazole and amphotericin B resulted in non-significant (*p* > 0.05) growth inhibition of mammalian cells in comparison to 0.5% DMSO-treated control cells. Thus, it appears that while the concentrations used in the combination study exhibited synergistic effects on *N. fowleri*, they were non-toxic to human kidney or intestinal cells.

## 4. Discussion

Over the past few years, we have investigated the steroidogenic pathway in free-living amoebae *N. fowleri* and *Acanthamoeba castellanii*, and we conducted studies with chemical inhibitors to validate several steroidogenic enzymes as drug targets [11,20,24,25,26]. We found that CYP51 inhibitors, posaconazole and itraconazole, are superior to amphotericin B, and isavuconazole is equipotent to amphotericin B against trophozoites of the *N. fowleri* KUL strain [11,20]. While *N. fowleri* trophozoites are susceptible to CYP51 inhibitors, most of the FDA-approved conazoles, including posaconazole and itraconazole, that exhibit potent anti-*Naegleria* activity in vitro are not brain-penetrant and, therefore, may not be viable candidates for the treatment of PAM [11]. CNS pharmacokinetics of posaconazole and itraconazole are similar to amphotericin B. None of the amphotericin B formulations produce measurable CSF concentrations regardless of inflammation in the CNS [27,28]. Similarly to amphotericin B, itraconazole and posaconazole CSF concentrations indicate poor BBB permeability. However, their successful use for fungal infections of the CNS may be due to the accumulation of drugs in brain tissue [29,30,31,32]. Brain parenchymal kinetics may also explain some in vivo efficacy of posaconazole in a mouse model of PAM (two of six mice cured at a 20 mg/kg dose) reported earlier [33]. But, intravenous formulations of posaconazole and itraconazole include cyclodextrin to enhance solubility, leading to nephrotoxicity [34].

Antifungal drugs known as azoles were introduced in 1944 and are characterized by their imidazole-based structures; they include the over-the-counter drug clotrimazole (Lotrimin^®^) used for athlete’s foot [35]. The transition from first- to second-generation azoles is marked by the replacement of the imidazole moiety with a triazole-based structure. Fluconazole (Diflucan^®^), a major influencer for this structural change, was introduced to the US market in 1989 and has since been indicated for vaginal candidiasis, oropharyngeal and esophageal candidiasis, and cryptococcal meningitis. The first reported use of fluconazole for the treatment of PAM was in Mexico in 2003; the patient was given intravenous fluconazole at 10 mg/kg/day every 24 h for 1 month [36]. Although fluconazole can distribute rapidly through tissues, including the CNS [37,38], it exhibited the lowest in vitro activity against *N. fowleri* [11].

**Figure 7:**
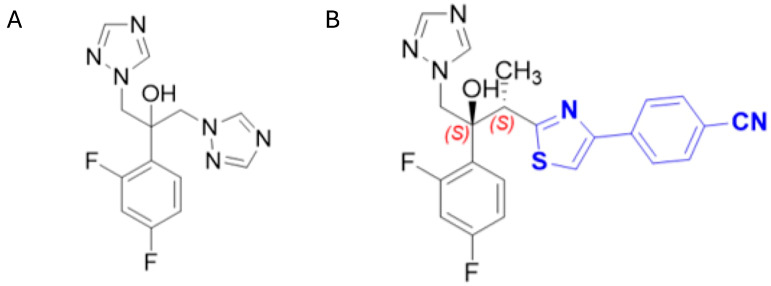
Structures of (**A**) fluconazole and (**B**) isavuconazole.

The development of isavuconazole carries many structural homages to fluconazole (Figure 7A) with some notable elements that improve upon its pharmacokinetic properties and antifungal activities. Isavuconazole (Figure 7B) contains two chiral centers denoted by the absolute stereochemistry (Figure 7B, red), as opposed to fluconazole which is notably achiral. In the fungal models, chirality in the aliphatic regions results in a specific conformation such that the aromatic side chain (Figure 7B, denoted in blue) moves antiperiplanar to the triazole ring, with the methyl group competing for the binding pocket of the C-13 position of lanosterol, thus inhibiting downstream sterol biosynthesis [39,40,41]. Isavuconazole is the metabolite of isavuconazonium (Cresemba^®^), which received FDA approval for the treatment of invasive aspergillosis and mucormycosis in 2015 [42].

The prodrug isavuconazonium exhibited mean EC_50_ values ranging from 0.1 (strain KUL) to 4.7 µM (CDC:V1005) (Table 1). Although there was a difference in the susceptibility of strains to isavuconazonium, the major metabolite isavuconazole is more clinically relevant than the prodrug. When the metabolite isavuconazole was tested on multiple strains, it demonstrated nanomolar potency on different strains, with EC_50_ values ranging between 0.1 and 0.6 µM (Table 1). Since butyrylcholinesterase that hydrolyzes isavuconazonium to active isavuconazole is present in the FBS [43] of assay conditions containing *N. fowleri* culture medium, it is possible that isavuconazonium, when tested for potency on *N. fowleri* trophozoites, metabolized in vitro to isavuconazole. But the amount of butyrylcholinesterase in FBS is much lower (0.04 µg/mL) than the concentration in human serum (4 µg/mL) [43], and the difference in the EC_50_ values of isavuconazonium and isavuconazole against different strains further reflected the activity of the prodrug itself.

Both isavuconazonium and isavuconazole were relatively fast-acting and they required 24 h to achieve nanomolar potency (Figure 3). The prodrug as well as the metabolite exhibited a synergistic effect on trophozoites when each compound was combined with the recommended drug amphotericin B (Table 3 and Table 4, and Figure 4 and Figure 5), but the synergistic combination did not elicit toxicity on mammalian kidney and intestinal cells.

The major advantages of the prodrug isavuconazonium sulfate over other conazoles include its water solubility, which eliminates the concerns of nephrotoxicity caused by cyclodextrin, and availability in intravenous and oral formulations to treat both pediatric and adult patients. The recommended dosage of 10 mg/kg intravenously once daily or 372 mg orally once daily for pediatric patients is well-tolerated with minimum adverse effects including diarrhea, nausea, vomiting, and headache. The drug is rapidly absorbed following oral administration with a bioavailability of 98% [15]. In healthy volunteers, the drug exhibited attractive pharmacokinetic characteristics like slow elimination and tissue distribution in a high volume [44,45].

In a published study [34] with isavuconazonium sulfate, a single oral dose was administered to mice at 256 mg/kg. Blood and brain samples were collected at 1, 3, 6, and 24 h postdosing and analyzed for isavuconazole concentration. An average of 5.2 µg/mL or about 12 µM isavuconazole was found circulating in the blood at 24 h after dosing. This concentration exceeded by about 20- to 100-fold the EC_50_ value of isavuconazole against *N. fowleri* in culture. Isavuconazonium was also found to be brain-penetrant as determined by the brain/plasma value of isavuconazole, which was 1.5 at 24 h postdosing [34]. In human studies, isavuconazole achieves a mean plasma concentration of 2.6 mg/L and it reaches maximum plasma concentration within 2–3 h following a single oral administration of 372 mg of isavuconazonium, which is equivalent to 200 mg of isavuconazole [15]. This plasma concentration is about 10- to 60-fold more than the EC_50_ value of isavuconazole against *N. fowleri*. Moreover, isavuconazole is extensively distributed throughout the tissues including the brain, with a mean steady state volume of distribution of about 450 L following intravenous administration [15]. In a study with three patients with coccidiodal meningitis, the CSF concentration of isavuconazole ranged from 0.45 to 1.72 µg/mL [46]. The distribution of the active metabolite in the brain and the in vitro activity of both the prodrug and the metabolite, either alone or in combination with amphotericin B, lend support to future investigation on the in vivo efficacy of isavuconazonium alone and in partnership with amphotericin B in an animal model of PAM.

## Figures and Tables

**Table 1:** EC_50_ values of isavuconazonium sulfate and isavuconazole against trophozoites of different *N. fowleri* strains.

Inhibitor	Strain	Mean EC_50_ (µM)	95% Lower CL (µM) ^a^	95% Upper CL (µM)
Isavuconazonium sulfate	KUL	0.1	0.1	0.1
	Davis	1.9	1.5	2.5
	CAMP	3.8	3.1	4.7
	CDC:V1005	4.7	3.5	6.3
Isavuconazole	KUL	0.1	0.1	0.1
	Davis	0.5	0.4	0.6
	CAMP	0.6	0.5	0.7
	CDC:V1005	0.6	0.5	0.8
**Standards of care**				
Amphotericin B [22]	KUL	0.2	0.2	0.2
	Davis	0.3	0.2	0.3
	CAMP	0.3	0.3	0.4
	CDC:V1005	0.5	0.4	0.6
Miltefosine [22]	KUL	54.5	51.4	57.8
	Davis	58.9	41.3	83.9
	CAMP	21.8	19.9	23.8
	CDC:V1005	15.9	10.3	24.7

^a^ CL—confidence limit.

**Table 2:** CC_50_ values of isavuconazonium sulfate and isavuconazole against two mammalian cells.

Inhibitor	Cells	Mean CC_50_ (µM)	95% Lower CL (µM)	95% Upper CL (µM)
Isavuconazonium sulfate	HEK293	15.9	13.4	18.7
	HT-29	39.7	35.7	44.2
Isavuconazole	HEK293	21.4	18.1	25.2
	HT-29	32	29.2	35.1
Amphotericin B	HEK293	17.4	15.4	19.7
	HT-29	6.2	5.1	7.6

**Table 3:** Synergistic effect of isavuconazonium sulfate and amphotericin B, shown for 97% growth inhibition of *N. fowleri* KUL trophozoites.

Isavuconazonium/Amphotericin B Ratio	% Growth Inhibition	Combination Index (CI)	Dose Reduction Index (DRI)	
			Isavuconazonium	Amphotericin B
2:1	97	0.4 ± 0.1	18.8 ± 11.8	3.2 ± 0.8
4:1	97	0.4 ± 0.1	13 ± 9.5	4.4 ± 1.6
8:1	97	0.4 ± 0.2	7 ± 4.9	4.7 ± 1.6

**Table 4:** Synergistic effect of isavuconazole and amphotericin B, shown for 97% growth inhibition of *N. fowleri* KUL trophozoites.

Isavuconazole/ Amphotericin B Ratio	% Growth Inhibition	Combination Index (CI)	Dose Reduction Index (DRI)	
			Isavuconazole	Amphotericin B
2:1	97	0.3 ± 0.2	373.7 ± 185.1	4.9 ± 3
4:1	97	0.2 ± 0.1	252.7 ± 141.3	6.6 ± 4.5
8:1	97	0.2 ± 0.1	170.5 ± 101.2	8.9 ± 6.4

## Data Availability

Data is contained within the article.

## Supplementary Materials

The following supporting information can be downloaded at https://www.mdpi.com/article/10.3390/pharmaceutics18010103/s1, Figure S1: EC_50_ curves of isavuconazole, dissolved in two different solvents, at 48 h against *N. fowleri*; Figure S2: The synergistic effect of isavuconazonium sulfate and amphotericin B, as analyzed by CompuSyn; Figure S3: The synergistic effect of isavuconazole and amphotericin B, as analyzed by CompuSyn.

## Abbreviations

The following abbreviations are used in this manuscript:

EC_50_	50% Effective Concentration
CC_50_	50% Cytotoxic Concentration
HEK	Human Embryonic Kidney
