# Supplementary material for: The Activity of FDA-Approved Prodrug Isavuconazonium Sulfate and Its Major Metabolite Isavuconazole Against Naegleria fowleri
Source: Pharmaceutics. 2026 Jan 12;18(1):103. doi: 10.3390/pharmaceutics18010103 (PMC12844783; doi:10.3390/pharmaceutics18010103)
Supplement: Supplementary file 1 [file pharmaceutics-18-00103-s001.zip › pharmaceutics-3929479-supplementary.pdf]

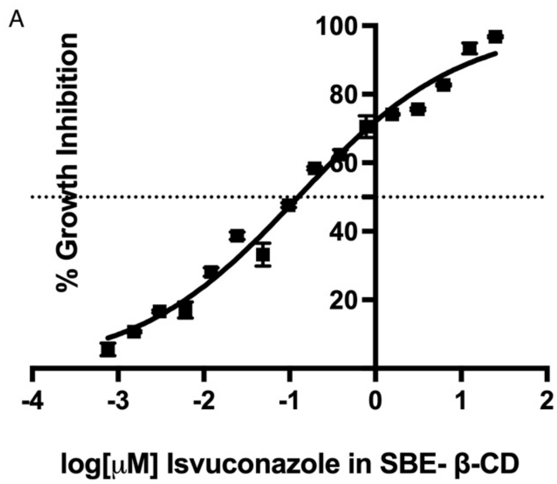

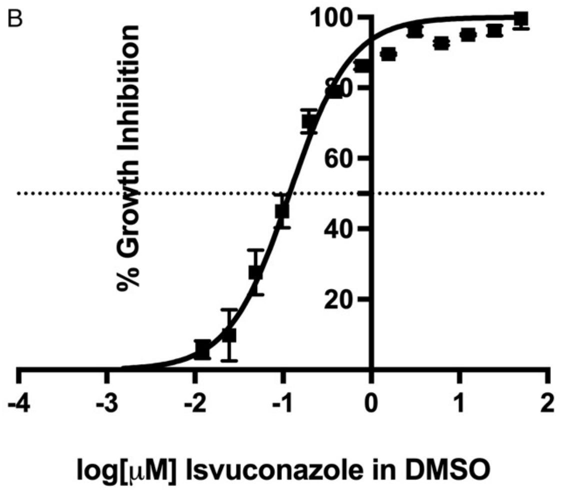

Figure S1. EC_{50} curves of isavuconazole, dissolved in two different solvents, at 48 h against *N. fowleri*. KUL trophozoites were treated with different concentrations of compounds in triplicate and growth inhibition curves were generated from mean ± standard error of mean (SEM) of (**A**) isavuconazole dissolved in 40% (w/v) SBE- β-CD and (**B**) isavuconazole dissolved in 100% DMSO. Dashed line represents 50% growth inhibition.

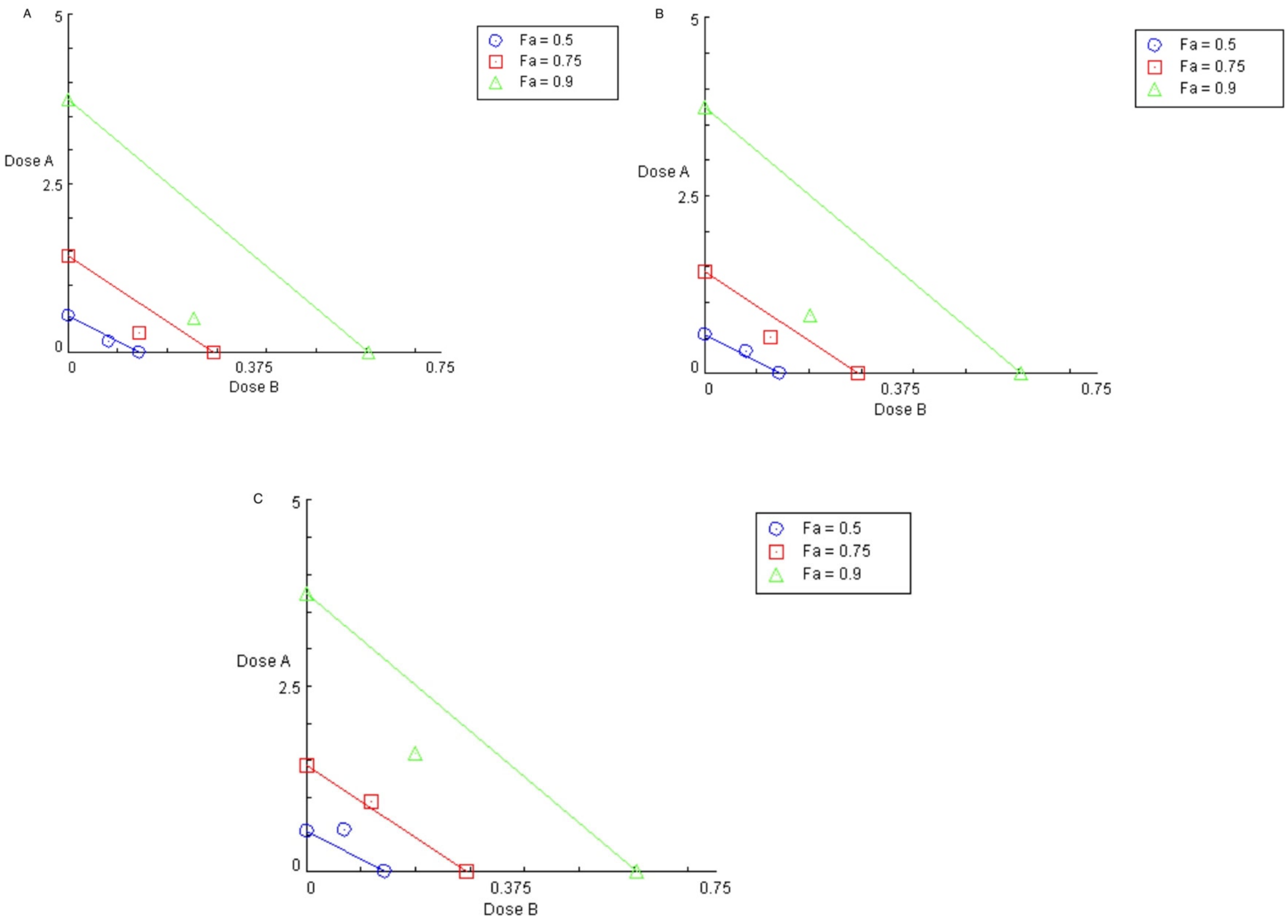

Figure S2. The synergistic effect of isavuconazonium sulfate and amphotericin B, as analyzed by CompuSyn. The isobolograms indicate effective doses required for each drug to induce 50% (Fa = 0.5), 75% (Fa = 0.75), and 90% (Fa = 0.9) growth inhibition of *N. fowleri* trophozoites at a combination of (**A**) 2:1, (**B**) 4:1, and (**C**) 8:1 ratio of isavuconazonium (Dose A) and amphotericin B (Dose B). Synergism is indicated by the pairing of the dose of each drug plotted as a point (symbol) below the respective Fa isobole or line.

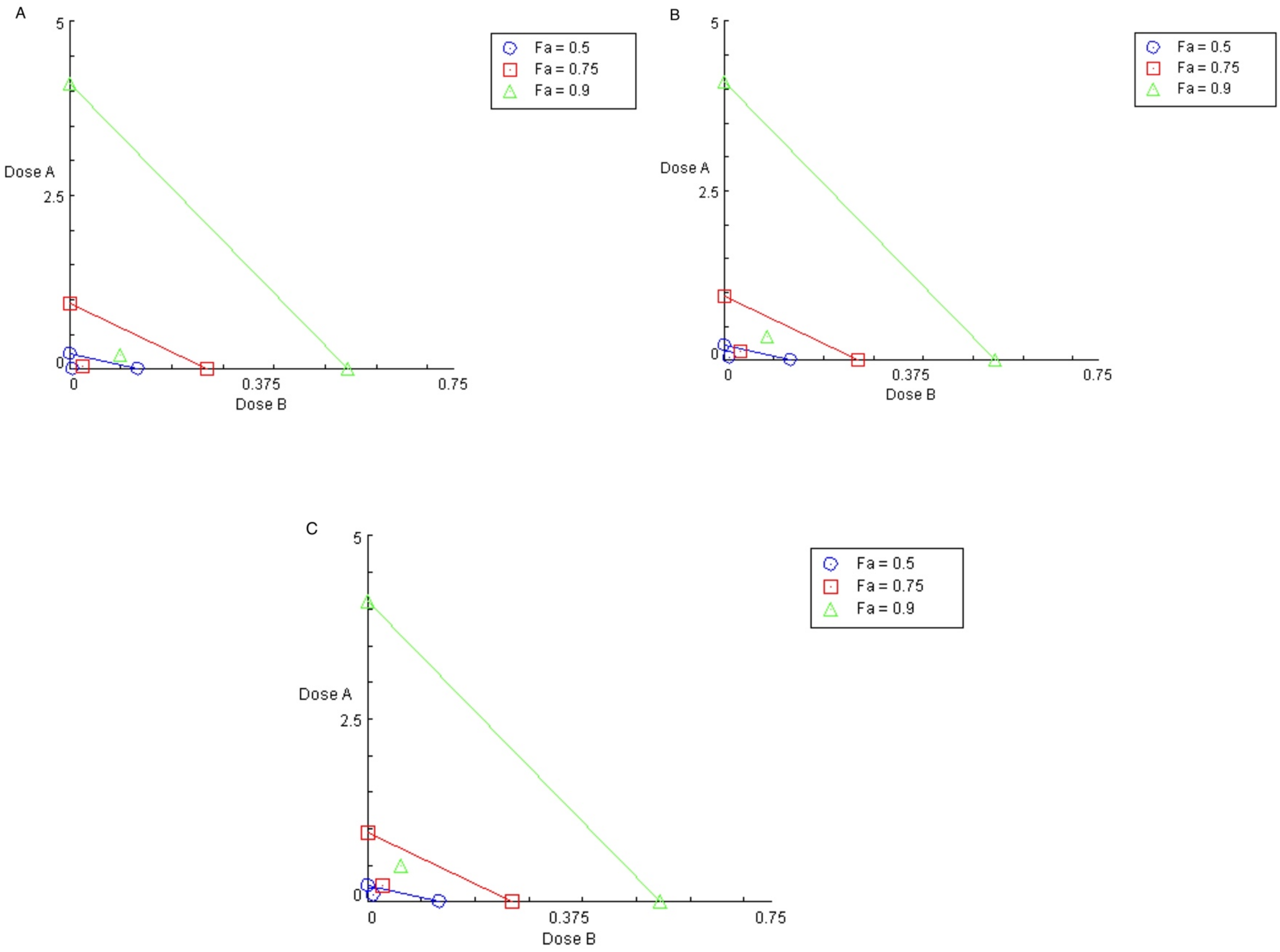

Figure S3. The synergistic effect of isavuconazole and amphotericin B, as analyzed by CompuSyn. The isobolograms indicate effective doses required for each drug to induce 50% (Fa = 0.5), 75% (Fa = 0.75), and 90% (Fa = 0.9) growth inhibition of *N. fowleri* trophozoites at a combination of (**A**) 2:1, (**B**) 4:1, and (**C**) 8:1 ratio of isavuconazole (Dose A) and amphotericin B (Dose B). Synergism is indicated by the pairing of the dose of each drug plotted as a point (symbol) below the respective Fa isobole or line.
